# Pitfalls in the surgical treatment of undiagnosed lung lesions and cystic pulmonary hydatidosis

**DOI:** 10.1186/s13019-022-02026-y

**Published:** 2022-10-27

**Authors:** Demet Yaldız, Güntuğ Batıhan, Kenan Can Ceylan, Sadık Yaldız, Seher Susam

**Affiliations:** 1 Faculty of Medicine, Manisa Celal Bayar University, Manisa, Turkey; 2 Kars State Hospital, Kars, Turkey; 3 Department of Thoracic Surgey, Dr Suat Seren Chest Diseases and Chest Surgery Training and Research Hospital, University of Health Sciences Turkey, Yenişehir, Gaziler Street 331, 35110 Izmir, Turkey

**Keywords:** Cavity, Diagnosis, Hydatid cyst, Imaging, Malignancy

## Abstract

**Background:**

Hydatid cysts can mimic many lung pathologies radiologically, as well as some malignant or benign lung tumors may show hydatid cyst-like radiological features. The aim of our study is to present our clinical experience and recommendations by analyzing the cases that create diagnostic difficulties by presenting a common radiological pattern with a pulmonary hydatid cyst.

**Methods:**

The patients who were operated on with a preliminary diagnosis of hydatid cyst but were diagnosed differently, and who were operated on with different prediagnoses and unexpectedly diagnosed with hydatid cyst were included in the study. The clinical and radiological features of the patients were documented, and the features of the cases that could cause difficulties in diagnosis and treatment for the surgeon were revealed.

**Results:**

A total of 20 patients who were radiologically suggestive of hydatid cyst but were diagnosed differently or unexpectedly diagnosed as hydatid cyst were included in the study. Lung cancer, bronchogenic cyst, or bronchiectasis were detected in 13 patients who were radiologically suggestive of hydatid cyst. There were 7 patients who were diagnosed with hydatid cysts, although they did not have specific radiological findings.

**Conclusions:**

While hydatid cysts can mimic many lung pathologies, many benign or malign parenchymal lung pathologies may exhibit hydatid cyst-like radiological features. Therefore, in regions where a hydatid cyst is endemic, the surgeon should consider all possibilities while managing the cases.

*Clinical registration number*: Institutional Review Board of the Dr Suat Seren Chest Diseases and Chest Surgery Education and Research Center (No. 49109414-604.02).

## Background

Hydatid cyst is a parasitic infection caused by Echinococcus granulosus that can affect many organs, especially the liver and lungs. Humans act as an accidental intermediate host, and the main transmission route is oral ingestion of egg-containing water or foods. After ingestion of eggs, they hatch in the small bowel and develop into oncospheres. Oncospheres, which penetrate the intestinal wall and enter the circulation, can migrate to several tissues, often the liver and lung. After the incubation period, oncospheres collect water around it and form the fluid-filled cyst [1–3]. In adults, liver localization (60–70%) is more common than lung (20–30%) localization [2, 3]. Radiological findings vary according to the size and number of the cyst and whether it is intact or not [4]. Therefore, hydatid cysts can mimic many lung pathologies radiologically, as well as some malignant or benign lung tumors may show hydatid cyst-like radiological features.

The aim of our study is to present our clinical experience and recommendations by analyzing the cases that create diagnostic difficulties by presenting a common radiological pattern with a pulmonary hydatid cyst.

## Methods

### Patient selection

Patients who were operated on for undiagnosed lung pathologies presenting radiologically cystic, cavitary, or heterogeneous opacity between January 2015 and January 2021 were analyzed retrospectively. The patients who were operated on with a preliminary diagnosis of hydatid cyst but were diagnosed differently (group 1), and who were operated on with different prediagnoses and unexpectedly diagnosed with hydatid cyst (group 2) were included in the study. The medical records of these patients were reviewed. Preoperative, operative and postoperative variables were recorded. Pulmonary lesions with a wall thickness of more than 4 mm in chest CT were considered cavities rather than cysts. All CT scans were reported by radiologists with at least 10 years of experience in the field.

Different criteria were used for patient selection for group 1 and group 2. For group 1, patients with cystic or smooth and thin-walled (< 1 cm) cavitary lesions radiologically suggestive of hydatid cyst were included in the study. All patients who were diagnosed with lung hydatid cyst after surgery were reviewed and patients without specific radiological findings in terms of hydatid cyst were included in the study as group 2 patients.

In case of clinically or radiologically active pulmonary infection, appropriate antibiotic therapy was administered before the operation.

Preoperative pulmonary evaluation including spirometry, diffusing capacity for carbon monoxide (DLCO) and, if necessary, VO2max and cardiopulmonary exercise tests was performed for each patient.

Chest radiogram, thorax computed tomography, and abdominal ultrasound was performed. The need for a PET-CT scan was evaluated considering the patient's age, lung cancer risk status, and the characteristics of the lesion on CT.

Transthoracic fine needle aspiration biopsy, which is contraindicated in cystic lesions, was also not preferred for patients with cavitary lesions due to their thin wall thickness. However, bronchoscopy was performed in all cases. Transthoracic fine-needle aspiration biopsy was performed in patients with heterogeneous opacity on thorax CT.

Due to its relatively low sensitivity in the diagnosis of lung hydatid cyst, anti-Echinococcus IgG ELISA test was not routinely performed, but it was preferred in cases with radiologically suspected hydatid cysts.

### Surgical technique

Video-assisted thoracic surgery (VATS) or muscle sparing posterolateral thoracotomy was performed. Although we do not have definite criteria for the choice of the surgical method, VATS was preferred more frequently in peripherical localized lesions smaller than 5 cm. Two or three port incisions were used in patients who underwent VATS.

In order to make the diagnosis intraoperatively, first of all, the cyst or cavity was aspirated, and the lesion wall was incised and explored for the laminated membrane. In the absence of the laminated membrane, the cyst-cavity contents and wall were sampled for extemporaneous examination and studied as frozen in order to make a definitive pathological diagnosis. Cyst excision, wedge resection, or lobectomy operations were performed according to the pathological evaluation and surgical exploration findings. Anatomical lung resection and systematic lymph node dissection were performed for lesions reported as malignant as a result of intraoperative frozen examination. Incomplete tests of staging in patients who were unexpectedly diagnosed with lung cancer were completed in the postoperative period after the patients were recruited.

### Postoperative follow-up

We have preferred to keep the patients in the intensive care unit (ICU) for the first 24 h after the operation. The expansion status of the lung was followed by a daily chest X-ray. Chest tubes were terminated in patients with expanded lungs and no active air or fluid drainage. After the chest tube removal, patients who were in stable condition and enabled self-maintenance of normal daily activities were discharged.

### Statistical analyzes

Our study was designed as a case series. Therefore, advanced statistical analysis methods were not used. Continuous variables are expressed as mean value ± standard deviation (SD). Non-normal distributed values were expressed as medians and interquartile ranges.

## Results

A total of 20 patients who were radiologically suggestive of hydatid cyst but were diagnosed differently or unexpectedly diagnosed as hydatid cyst were included in the study. There were 11 men and 9 women. The median age was 48 years, with a range of 20–73.

**Fig. 1:**
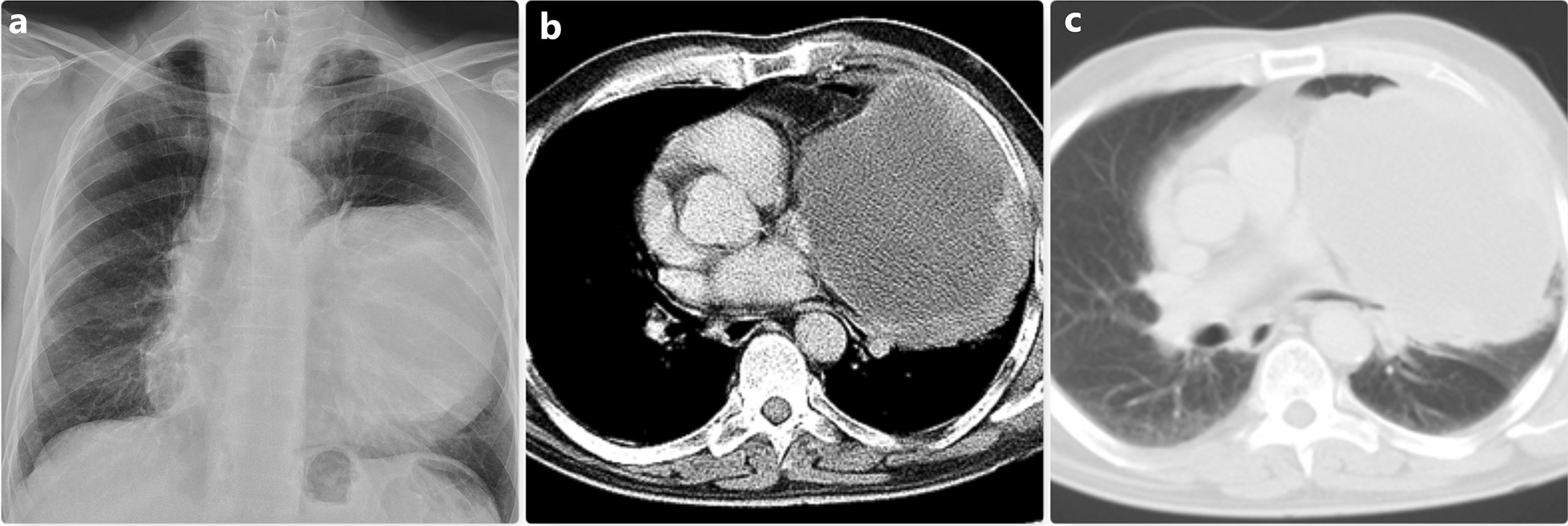
The left-sided cavity was seen in the chest radiogram (**a**) On thorax CT, a giant cavitary lesion pushing the mediastinum to the opposite side is observed (**b** and **c**) In this patient, the diagnosis was made as large cell carcinoma

**Fig. 2:**
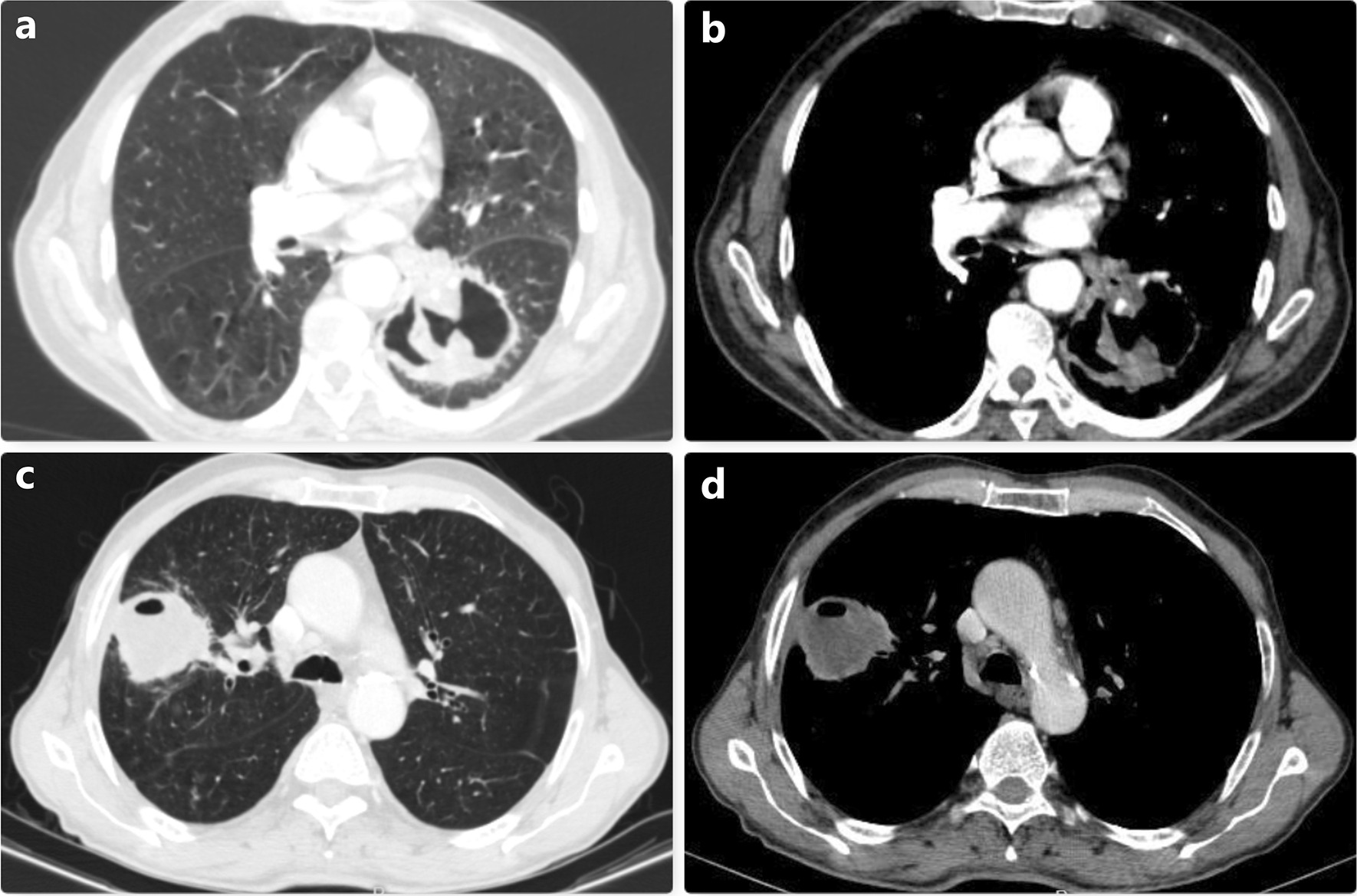
In (**a**) and (**b**), thorax CT images of the cavitary lesion located in the left lower lobe are observed. In this patient who was operated with a preliminary diagnosis of hydatid cyst, the diagnosis was made as squamous cell carcinoma and lobectomy was performed. (**c**) and (**d**) show a cavitary lesion located in the right upper lobe. In this patient, squamous cell lung cancer was diagnosed intraoperatively, and upper lobectomy was performed

Lung cancer, bronchogenic cyst, or bronchiectasis were detected in 13 patients who were radiologically suggestive of hydatid cyst (Group 1). Of the 5 patients who were operated on for a cavitary lesion and were diagnosed with lung cancer, 4 had squamous and 1 had large cell carcinoma histology (Figs. 1 and 2). The most common symptom in this group of patients was non-productive cough. One patient underwent emergency operation due to massive hemoptysis. The most frequently preferred surgical approach was thoracotomy, and the type of resection performed was lobectomy. While no intraoperative complications were observed, postoperative complications were seen in 4 patients (Table 1).

**Table 1:** Characteristics of the group 1 patients

No	Sex	Age	Symptom	Side	CT features	IgG ELISA*	Surgical approach	Operation	Diagnosis	Postoperative complication	Drainage time (day)
1	M	73	Hemoptysis	Left	Cavitary**	N/A	Thoracotomy	Lobectomy	Squamous cell ca	Pneumonia	4
2	M	67	Cough	Right	Cavitary**	N/A	Thoracotomy	Lobectomy	Squamous cell ca	Pneumonia	12
3	M	55	None	Right	Cavitary**	–	VATS	Lobectomy	Squamous cell ca	PAL	8
4	M	64	None	Left	Cavitary**	–	Thoracotomy	Lobectomy	Large cell ca	PAL	8
5	M	70	Massive hemoptysis	Left	Cavitary**	N/A	Thoracotomy	Pneumonectomy	Squamous cell ca	None	3
6	M	30	Chest pain	Right	Cystic	–	Thoracotomy	Cyst excision	Bronchogenic cyst	None	5
7	F	50	Chest pain	Right	Cystic	–	Thoracotomy	Cyst excision	Bronchogenic cyst	None	5
8	M	37	Cough	Right	Cystic	–	VATS	Cyst excision	Bronchogenic cyst	None	6
9	M	20	Cough	Left	Cystic	N/A	Thoracotomy	Lobectomy	Bronchogenic cyst	None	5
10	F	27	Cough	Right	Cavitary**	N/A	Thoracotomy	Lobectomy	Bronchogenic cyst	None	5
11	F	38	Hemoptysis	Left	Cavitary**	N/A	Thoracotomy	Lobectomy	Bronchiectasis	None	4
12	F	21	Cough	Right	Cystic	–	VATS	Lobectomy	Bronchiectasis	None	4
13	M	46	Chest pain	Right	Cystic	N/A	Thoracotomy	Wedge	Glomangioma	None	4

*M* Male, *F* Female, *CT* Computed tomography, *N/A* Not applicable, *PAL* persistent air leak, *VATS* Video-assisted thoracic surgery. *Anti-Echinococcus IgG ELISA. **The cavity wall of these lesions was thin and relatively regular

**Fig. 3:**
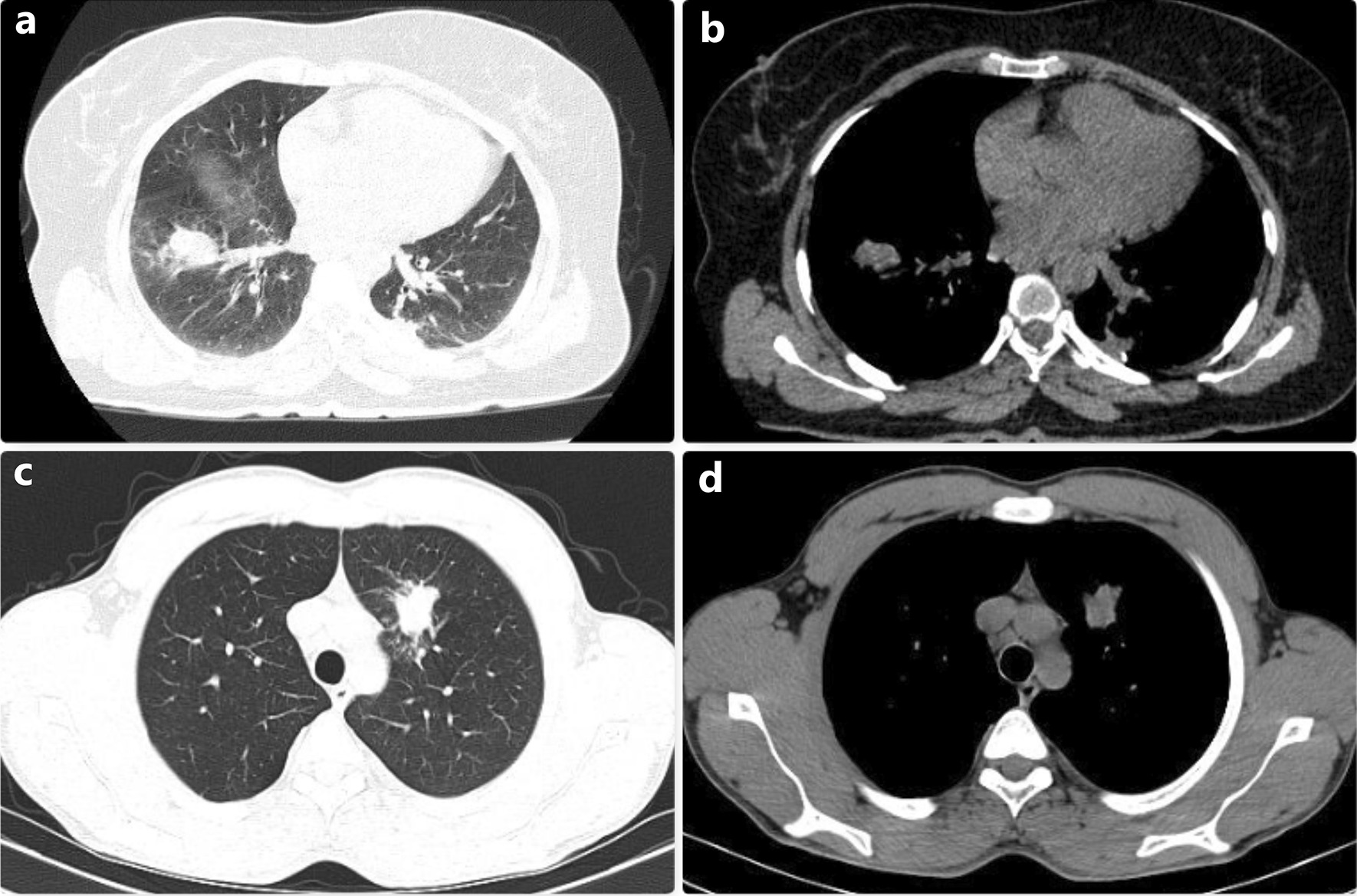
Thoracic CT images of two patients who were operated with a preliminary diagnosis of lung cancer due to heterogeneous opacity in the lung are observed. As a result of the biopsies performed during the operation, it was identified that these images belonged to the perforated hydatid cyst

**Fig. 4:**
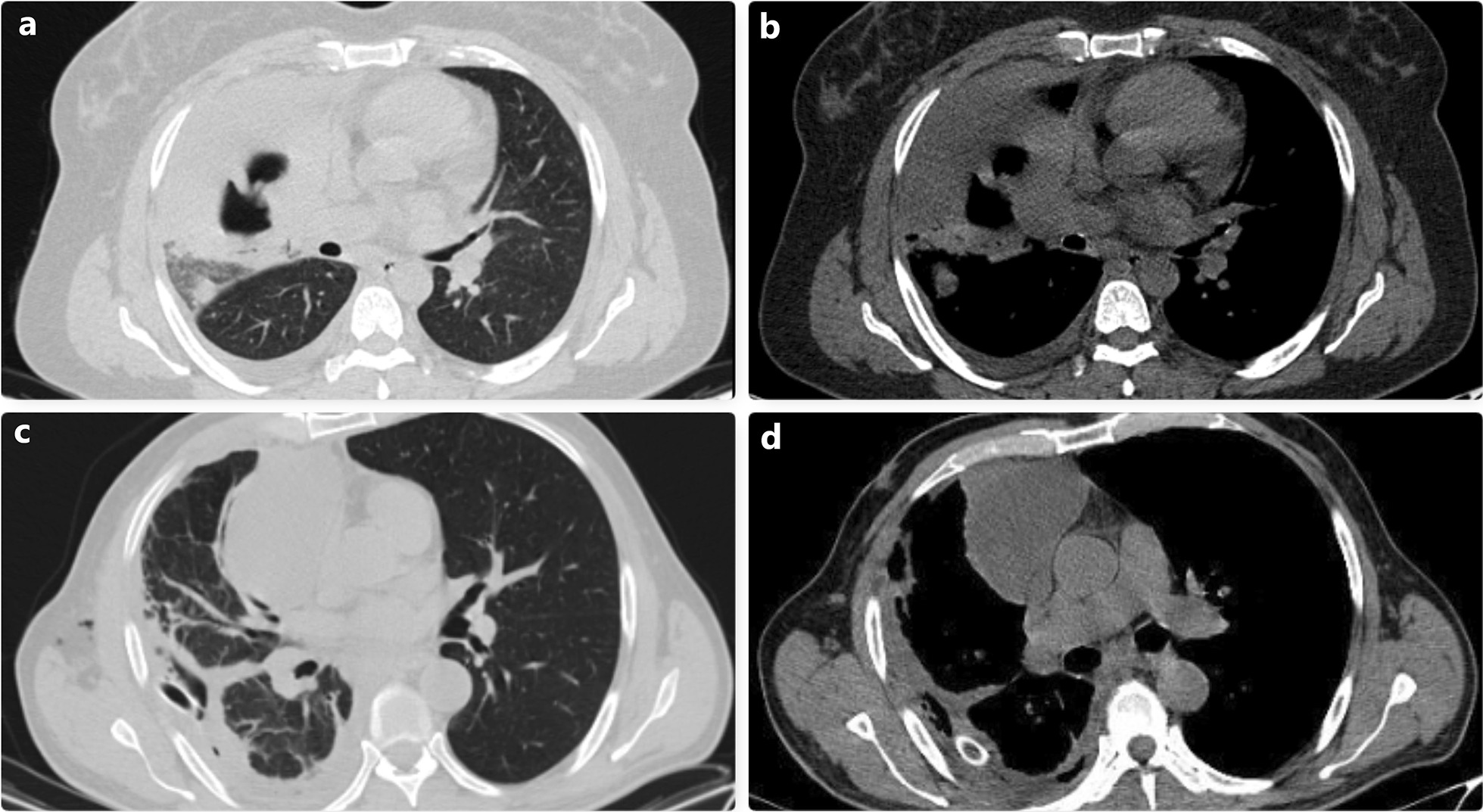
Thoracic CT images of two different patients who were followed up with the diagnosis of empyema are shown. The operation performed due to the persistence of the infection status and loculation despite medical treatment revealed that these cases had complicated hydatid cysts

There were 7 patients who were diagnosed with hydatid cysts, although they did not have specific radiological findings (Group 2). Two of these patients had increased heterogeneous parenchymal opacities and radiologically suggested lung cancer (Fig. 3). Two patients had loculated pleural effusion with pleural thickening (Fig. 4) and the other three patients had a cavitary lesion with a thick and irregular wall. Non-productive cough is also the most common symptom in this group. Bronchoscopy was performed in all patients during the preoperative period, and transthoracic fine needle aspiration biopsy was performed in two patients with heterogeneous parenchymal opacity, but preoperative histological diagnosis could not be made in any of the patients. Cystotomy plus capitonnage was performed in four of the 7 patients diagnosed with hydatid cyst, and wedge resection was performed in the other three patients. No intraoperative and postoperative complications were observed. The clinical and radiological data of the patients in this group are shared in detail in Table 2.

**Table 2:** Characteristics of the group 2 patients

No	Sex	Age	Symptom	Side	CT features	Liver localization	Surgical approach	Operation	Diagnosis	Postoperative complication	Drainage time (day)
1	F	42	Chest pain	Left	Cavitary	None	VATS	Wedge	Hydatid cyst	None	3
2	F	64	None	Left	Cavitary	None	VATS	Wedge	Hydatid cyst	None	3
3	F	35	Hemoptysis	Left	Cavitary	Yes	Thoracotomy	Cystotomy plus capitonnage	Hydatid cyst	None	4
4	M	38	Cough	Right	Heterogeneous opacity	None	Thoracotomy	Cystotomy plus capitonnage	Hydatid cyst	None	2
5	M	52	Cough	Left	Heterogeneous opacity	None	Thoracotomy	Wedge	Hydatid cyst	None	2
6	F	51	None	Right	Loculated pleural effusion	None	Thoracotomy	Cystotomy plus capitonnage + decortication	Hydatid cyst + ampyema	None	4
7	M	66	None	Right	Loculated pleural effusion	Yes	Thoracotomy	Cystotomy plus capitonnage + decortication	Hydatid cyst + ampyema	None	5

*M* Male, *F* Female, *CT* Computed tomography, *VATS* Video-assisted thoracic surgery

When all cases are analyzed together the mean drainage time was found as 4.7 ± 2.2 days, and the mean hospital stay was found as 6.3 ± 2.8 days. No operative and postoperative mortality were observed.

## Discussion

Hydatid cyst also known as cystic echinococcosis is endemic in the Middle East, Mediterranean countries, India, Africa, South America, New Zealand, and Australia. The annual incidence rate of cystic echinococcosis worldwide ranges from 1 to 200 cases per 100,000 inhabitants [1–3, 5]. The increase in the frequency of hydatid cysts up to 200 times in endemic regions makes it necessary to keep cystic echinococcosis in mind in the differential diagnosis of lung and liver pathologies in these regions.

Although some specific radiological findings have been described in the literature, it is not always easy to radiologically diagnose pulmonary cyst hydatid [6–8]. Particularly, the radiological findings of complicated hydatid cysts are extremely diverse. While uncomplicated hydatid cysts present smooth, hyperdense cyst walls and well-circumscribed fluid attenuation radiological findings are extremely heterogeneous in complicated ones [4, 8]. Therefore, hydatid cysts can be confused with benign parenchymal lesions (like infective lung lesions, bronchiectasis, and congenital lung malformations) or malignant diseases. This makes it necessary to consider hydatid cyst in the differential diagnosis of all cystic lung lesions.

Because of the elasticity of the lungs and intrapleural negative pressure, lung hydatid cysts tend to grow rapidly, and, in some patients, they can reach gigantic sizes. The risk of perforation of the hydatid cyst is directly proportional to its size. The radiological signs of the perforation in hydatid cysts have been named in different ways. The most frequently used of these are [9, 10]: inverse crescent sign, air bubble sign, ring enhancement sign, Cumbo sign, serpent sign, water lily sign and incarcerated membrane sign. These expressions are the names given to the radiological findings of different stages of hydatid cyst perforation. However, perforated hydatid cysts may not have any of the specific radiological signs mentioned above and may present with radiological findings that mimic very different lung pathologies.

Surgical treatment is curative in most cases of pulmonary hydatid cysts. Although different surgical techniques have been described in its treatment, cystotomy plus capitonnage is the most preferred method. The patient should be carefully evaluated in terms of possible differential diagnoses in the preoperative period in order to make the appropriate preoperative preparation and to choose the correct surgical technique [11–13].

Especially when hydatid cysts of the lung become complicated after perforation, they may present as thickening and irregularity in the cyst wall, or as parenchymal opacities.

In malignant lesions, the cavity wall is usually thicker and irregular [14, 15]. However, although rare, lung cancer may present with thin-walled cavitary lesions [16]. In regions where hydatid cyst is endemic, it is not easy to distinguish between complicated hydatid cyst and lung cancer during the preoperative period. The incidence of cavitation in primary lung cancers varies between 2 and 25%. The lung cancer types in which cavitary parenchymal lesions are most frequently observed are squamous cell lung cancer, adenocarcinoma, and large cell carcinoma, respectively [14, 15, 17, 18].

In our study, there were five patients with lung cancer radiologically mimicking complicated hydatid cysts. The histological subtype was squamous cell carcinoma in four, and large cell carcinoma in one patient. In these cases, the cavity wall was thin and relatively regular. All of these patients underwent bronchoscopy in the preoperative period, but no pathological diagnosis could be made. One of the most important problems in the management of patients radiologically mimicking complicated hydatid cysts is the risk of incomplete preoperative staging. Therefore, we recommend performing bronchoscopy in all patients presenting with a cavitary lung lesion, evaluating the radiological features of the cavity by an experienced radiologist, and investigating the lung cancer risk characteristics of the patient. As in our study, if a preoperative diagnosis cannot be made, pathological studies should be performed in the intraoperative period.

Perforated hydatid cysts may present only with heterogeneous increased parenchymal opacity without displaying cystic or cavitary features on thorax CT. Considering the possibility of lung cancer in patients with increased parenchymal opacity that cannot be regressed with antibiotic treatment, advanced diagnostic interventions such as transthoracic fine needle aspiration biopsy, brain MRI and PET-CT may be required. In our study, further investigation was performed with a preliminary diagnosis of lung cancer in two patients with heterogeneous parenchymal opacity increase that did not regress with antibiotic therapy, but surprisingly, a hydatid cyst was diagnosed in the intraoperative period. It is possible to encounter similar cases in the literature [19–21]. Çobanoglu et al. [21] reported clinical and radiological features of seven patients with tumors mimicking hydatid cyst and emphasized the diagnostic difficulties due to the variety of radiological features that hydatid cyst may show.

The perforated hydatid cyst may become infected and form an abscess, displaying a common radiological pattern with infective lung lesions [22, 23]. Surgery is indicated for lung lesions that persist despite medical treatment or in the case of recurring abscesses. In our study, a lung abscess associated with bronchiectasis was detected in two patients radiologically mimicking complicated hydatid cysts. Recognizing the clinical and biochemical signs of infection and arranging appropriate medical treatment will prevent unnecessary surgical interventions. It may be more difficult to elucidate the etiology in cases where empyema develops by opening the abscess cavity into the pleural space. In a study by Aribas et al. [24], 145 patients hospitalized for hydatid cysts were reviewed and pleural complications were detected in 40 patients. They found that empyema was to be one of the most common pleural complications with a rate of 7.6%.

We included two patients who underwent tube thoracostomy with the diagnosis of empyema and were diagnosed with hydatid cyst after surgical treatment due to prolonged air leak and suspected parenchymal opacities in the follow-ups. Our recommendation in cases of empyema is the careful evaluation of the lung parenchyma with thorax CT after the drainage of the purulent pleural contents and appropriate antibiotic therapy. In the presence of persistent cavitary or cystic parenchymal lesion despite pleural drainage and appropriate medical treatment, the possibility of a complicated hydatid cyst should be kept in mind.

Another group of diseases that may cause difficulties in the differential diagnosis is congenital lung malformations which represent a broad spectrum of pathology affecting the airway or lung parenchyma [25]. The most common congenital lung malformations are bronchogenic cyst, congenital cystic adenomatoid malformation, pulmonary sequestrations, and congenital lobar emphysema. Lesions may present radiologically as a single thin- or thick-walled cyst, multicystic, or solid lesions with heterogeneous density [25, 26]. Cysts may be filled with air or fluid. These radiological findings of congenital lung malformations show great similarities with pulmonary hydatid cysts. In our study, intraparenchymal bronchogenic cysts were detected in four patients who radiologically suggested the diagnosis of pulmonary hydatid cysts with their thin-walled cystic lesions. In these cases, the absence of laminated membrane in the cyst during surgery and the revealing presence of cartilage tissue in the cyst wall in the frozen section enabled the diagnosis of bronchogenic cyst. Making a definitive diagnosis during the operation is very important in terms of directly affecting the surgical technique to be applied.

## Conclusions

Although many radiological features specific to pulmonary hydatid cyst have been defined, these features cannot be observed in many cases. While hydatid cysts can mimic many lung pathologies, many benign or malign parenchymal lung pathologies may exhibit hydatid cyst-like radiological features. Therefore, in regions where a hydatid cyst is endemic, the surgeon should consider all possibilities while managing the cases.

## Data Availability

The data underlying this article cannot be shared publicly due to the privacy of individuals that participated in the study. The data will be shared on reasonable request to the corresponding author.

## Abbreviations

CT: Computed tomography
DLCO: Diffusing capacity of the lungs for carbon monoxide
MRI: Magnetic resonance imaging
PET: Positron emission tomography
VATS: Video-assisted thoracic surgery
VO_2_max: Maximal oxygen consumption
